# Luftverschmutzung als wichtiger Kofaktor bei COVID-19-Sterbefällen

**DOI:** 10.1007/s12181-021-00508-2

**Published:** 2021-09-17

**Authors:** Thomas Münzel, Omar Hahad, Andrea Pozzer, Jos Lelieveld

**Affiliations:** 1 Zentrum für Kardiologie – Kardiologie I, Universitätsmedizin der Johannes Gutenberg-Universität Mainz, Langenbeckstr. 1, 55131 Mainz, Deutschland; 2 Max-Planck-Institut für Chemie, Abteilung Atmosphärenchemie, Johannes Gutenberg-Universität Mainz, Mainz, Deutschland

**Keywords:** Feinstaub, SARS-CoV‑2, Mortalität, Endotheliale Dysfunktion, Emissionen, Particulate matter, SARS-CoV‑2, Mortality, Endothelial dysfunction, Emissions

## Abstract

**Hintergrund:**

Die Sterblichkeit bei COVID-19 ist in Anwesenheit kardiopulmonaler Komorbiditäten erhöht. Luftverschmutzung ist ebenfalls mit einer erhöhten Sterblichkeit assoziiert, v. a. vermittelt durch kardiopulmonale Erkrankungen. Beobachtungen zu Beginn der COVID-19-Pandemie zeigten, dass die Sterblichkeit bei COVID-19 v. a. in Regionen mit stärkerer Luftverschmutzung erhöht ist. Ungeklärt ist der Einfluss von Luftverschmutzung für den Krankheitsverlauf bei COVID-19.

**Methode:**

Es wurde eine selektive Literaturrecherche von Studien bis Anfang April 2021 in PubMed zum Zusammenhang von Luftverschmutzung und der COVID-19-Mortalität mit den Suchbegriffen „air pollution AND/OR COVID-19/coronavirus/SARS-CoV‑2 AND/OR mortality“ durchgeführt.

**Ergebnisse:**

Aktuelle Untersuchungen belegen, dass etwa 15 % der weltweiten COVID-19-Todesfälle auf Luftverschmutzung zurückzuführen sind. Der Anteil der luftverschmutzungsbedingten COVID-19-Todesfälle in Europa liegt bei 19 %, in Nordamerika bei 17 % und in Ostasien bei 27 %. Diese Beteiligung der Luftverschmutzung an COVID-19-Todesfällen wurde mittlerweile ebenfalls durch verschiedene Studien aus den USA, Italien und England bestätigt. Luftverschmutzung und COVID-19 führen zu ähnlichen Schäden für das kardiopulmonale System, die möglicherweise den Zusammenhang zwischen Luftverschmutzung und erhöhter COVID-19-Mortalität erklären.

**Schlussfolgerung:**

Der hier gezeigte Umweltaspekt der COVID-19-Pandemie verlangt danach, dass man verstärkt nach wirksamen Maßnahmen zur Reduzierung anthropogener Emissionen, die sowohl Luftverschmutzung als auch den Klimawandel verursachen, streben sollte.

## Abstract (en)

**Background:**

The mortality from COVID-19 is increased in the presence of cardiopulmonary comorbidities. Air pollution is also associated with increased mortality, primarily mediated by cardiopulmonary diseases. Observations at the beginning of the COVID-19 pandemic showed that mortality from COVID-19 was increased, especially in regions with higher levels of air pollution. The influence of air pollution on the course of the disease in COVID-19 is unclear.

**Method:**

A selective literature search of studies up to the beginning of April 2021 was carried out in PubMed on the association between air pollution and COVID-19 mortality using the search terms “air pollution AND/OR COVID-19/coronavirus/SARS-CoV‑2 AND/OR mortality”.

**Results:**

Current research shows that around 15% of global COVID-19 deaths are due to air pollution. The proportion of COVID-19 deaths from air pollution is 19% in Europe, 17% in North America and 27% in East Asia. This role of air pollution in COVID-19 deaths has now been confirmed by various studies from the USA, Italy and England. Air pollution and COVID-19 result in similar damage to the cardiopulmonary system, which may explain the link between air pollution and increased COVID-19 mortality.

**Conclusion:**

The environmental aspect of the COVID-19 pandemic shown here calls for greater efforts to be made towards effective measures to reduce anthropogenic emissions, which cause both air pollution and climate change.

Schon im Rahmen der Spanischen Grippe wurde festgestellt, dass in Gebieten mit hoher Luftverschmutzung mehr Todesfälle im Rahmen von Influenzainfektionen zu beobachten waren [1]. Während der ersten SARS(Schweres Akutes Respiratorisches Syndrom)-Pandemie haben Cui et al. [2] eine ökologische Studie zu dem Thema Luftverschmutzung und Todesfälle aufgrund von SARS in der Volksrepublik China präsentiert. Hierbei stellten die Autoren fest, dass ein positiver Zusammenhang zwischen der Luftverschmutzung und den SARS-Todesfällen in der chinesischen Bevölkerung besteht. Insgesamt hatten bei einem moderat erhöhten Luftverschmutzungsindex infizierte Patienten ein um etwa 84 % erhöhtes Risiko, an SARS zu versterben [2].

Eine wichtige weltweit gemachte Beobachtung während der ersten Welle der COVID-19-Pandemie war eine enorme Verringerung der Aufnahme von Patienten mit einem akuten Koronarsyndrom in Krankenhäusern [3]. Ähnliches wurde für Patienten mit einer Herzinsuffizienz und Herzrhythmusstörungen wie Vorhofflimmern beobachtet [3]. Dieses Phänomen ist möglicherweise auf 4 Ursachen zurückzuführen:

- die Angst vor einer Ansteckung in Krankenhäusern,
- ein entspannter Lebensstil aufgrund geringerer Belastungen während des Corona-Lockdowns,
- eine Erhöhung der Schmerzschwelle (bei Herzschmerzen),
- eine erhebliche Verringerung der Luftverschmutzung.

Die mittlerweile beobachtete drastische Reduktion der Zahl der Herzinfarkte im Rahmen der COVID-19-Pandemie bei gleichzeitiger deutlicher Verbesserung der weltweiten Luftqualität [4] deutet indirekt darauf hin, dass Luftverschmutzung, was ebenfalls schon im Rahmen vorheriger Untersuchung nachgewiesen worden war, akut Herzinfarkte triggern kann und insgesamt die Prognose von Patienten mit Herzerkrankungen verschlechtern kann [3]. Das Ziel der vorliegenden Arbeit bestand darin, einen Überblick über Studien zum Zusammenhang von Luftverschmutzung und der COVID-19-Mortalität zu geben und zugrunde liegende pathophysiologische Mechanismen zu identifizieren.

## Methodik

Es wurde eine selektive Literaturrecherche von Studien bis Anfang April 2021 in PubMed zum Zusammenhang von Luftverschmutzung und der COVID-19-Mortalität auf Basis der klinisch-wissenschaftlichen Expertise der Autoren mit den Suchbegriffen „air pollution AND/OR COVID-19/coronavirus/SARS-CoV‑2 AND/OR mortality“ durchgeführt.

## COVID-19-Mortalität und Luftverschmutzung

Die Mortalität bei COVID-19 ist im Wesentlichen abhängig von Komorbiditäten, einschließlich von Erkrankungen bzw. kardiovaskulären Risikofaktoren wie Diabetes mellitus, arterielle Hypertonie, Fettleibigkeit und Rauchen [5]. Eine erhöhte COVID-19-Mortalität ist auch mit vermehrten kardiovaskulären Komplikationen wie einem Myokardinfarkt, Herzinsuffizienz, Herzrhythmusstörung und einem Anstieg von Risikomarkern wie dem Troponin verbunden, die interessanterweise auch im Zusammenhang mit Luftverschmutzung stehen [5]. Der Zusammenhang von Luftverschmutzung und der Mortalität bei COVID-19 ist daher naheliegend. Eine kürzlich erschienene Studie aus den USA ermittelte eine 11 % höhere Mortalitätsrate bei COVID-19 pro Anstieg von PM_2,5_ (Feinstaub mit einem aerodynamischen Durchmesser kleiner als 2,5 µm) um 1 µg/m^3^ in der Umgebungsluft [6].

**Figure 1:**
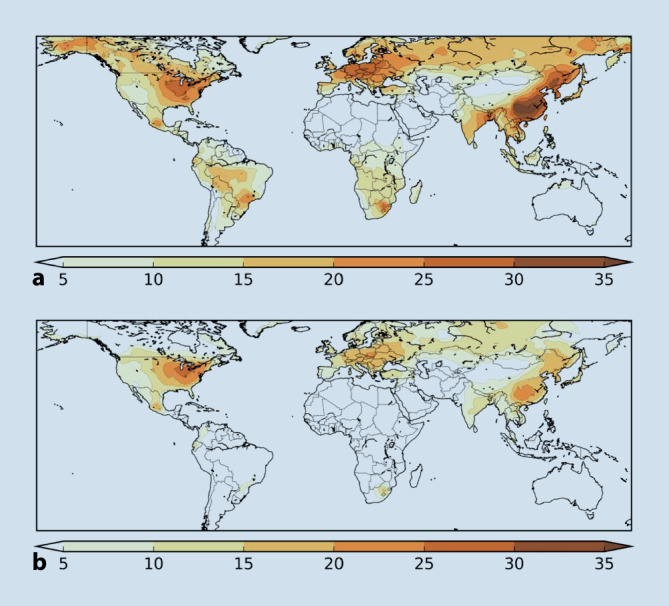

Pozzer und Lelieveld et al. haben kürzlich die Feinstaubkonzentrationen aufgrund von Satellitendaten, Luftverschmutzungsnetzwerken sowie mathematischen Modellen bestimmt und berechneten zudem den anthropogenen Anteil mit einem atmosphärischen Chemiemodell [7]. Das Ausmaß, in dem die Luftverschmutzung die COVID-19-Mortalität beeinflusst, wurde aus epidemiologischen Daten in den USA und China abgeleitet [2, 6]. Die Autoren schätzten, dass etwa 15 % der weltweiten COVID-19-Todesfälle auf Luftverschmutzung zurückzuführen sind, wobei dieser Anteil in Ostasien bei 27 % liegt, in Europa bei 19 % und in Nordamerika bei 17 %. Weltweit beziehen sich 50–60 % des zurechenbaren anthropogenen Anteils auf den Verbrauch fossiler Brennstoffe, in Europa, Westasien und Nordamerika liegt der Anteil bei 70–80 % (Abb. 1). Die Autoren schlussfolgerten, dass Luftverschmutzung das Mortalitätsrisiko bei COVID-19 signifikant erhöhen kann [7]. Aktuelle Studien aus Italien [8, 9] und England [10] konnten ebenfalls einen signifikanten Zusammenhang zwischen der Luftverschmutzung und der COVID-19-Mortalität bzw. einer -Infektion bestätigen. Zu konservativeren Ergebnissen kam eine Studie aus England mit Einschluss von 38.573 COVID-19-Todesfällen, die den Anteil für luftverschmutzungsbedingte COVID-19-Todesfälle auf 0,5 % bzw. 1,4 % pro Anstieg von NO_2_ (Stickstoffdioxid) bzw. PM_2,5_ um 1 µg/m^3^ bezifferte [11]. Dagegen ergab eine Analyse aus Norditalien eine um 11 % gestiegene Mortalitätsrate bei COVID-19 pro Anstieg um eine Einheit der PM_2,5_-Konzentrationen [12]. Ein weitere landesweite Studie aus Italien zeigte eine erhöhte Mortalitätsrate während der COVID-19-Pandemie in Abhängigkeit von den PM_2,5_-_,_ PM_2,5-10_- und PM_10_-Konzentrationen als im Vergleich zu präpandemischen Zeiten (2015 bis 2019) [13]. Eine gepoolte Analyse von COVID-19-Todesfällen aus 9 asiatischen Städten in Indien, Pakistan, Indonesien und China ergab eine signifikante Korrelation zwischen der PM_2,5_-Konzentration und der COVID-19-Mortaltiät, wobei der Zusammenhang für die PM_10_-Exposition schwächer ausfiel [14]. Eine weitere Studie aus China (Wuhan) konnte den Einfluss von PM_2,5_ und PM_10_ im Hinblick auf das COVID-19-Mortalitätsrisiko bestätigen [15]. Dagegen ergab eine Studie aus den USA (New York) zwar einen signifikanten Einfluss der Ozon- und PM_2,5_-Konzentrationen in Bezug auf das Risiko einer COVID-19-Infektion, jedoch konnte kein Zusammenhang mit der COVID-19-Mortalität beobachtet werden [16]. Eine Studie von 33 europäischen Ländern konnte signifikante Korrelationen zwischen der Konzentration verschiedener Luftschadstoffe (darunter PM_2,5_ und PM_10_) und den COVID-19-Sterbefällen sowie Infektionen ermitteln [17].

## Verschlechterte Prognose bei Patienten mit COVID-19 in Abhängigkeit von Luftverschmutzung

Die Luftverschmutzung entsteht in städtischen Umgebungen und besteht in der Regel aus Gasen oder Dampfphasenverbindungen und sekundären Schadstoffen einschließlich Stickstoffdioxid (NO_2_), flüchtigen organischen Verbindungen (einschließlich Benzol), Schwefeldioxid (SO_2_) und Ammoniak (NH_3_). Verbrennungsaerosole können grobe (PM_10_, Durchmesser < 10 µm), feine (PM_2,5_ < 2,5 µm) und ultrafeine Partikel (PM_0,1_ < 0,1 µm) enthalten. Während ein Feinstaubpartikel der Größe 10 µm etwa der Größe einer Zelle entspricht, haben die ultrafeinen Partikel (0,1 µm) die Größe eines Virus [18]. Je kleiner der Partikel ist, desto wahrscheinlicher ist es, dass er nach Inhalation die Lungen bzw. das Lungenepithel der Alveolen durchdringen kann (Transmigration), wodurch er in den Blutkreislauf gelangt und anschließend von der Gefäßwand aufgenommen wird. In der Gefäßwand stimuliert der Feinstaub Entzündungsprozesse, beschleunigt die Formation von oxidativem Stress im Gefäßsystem durch eine vermehrte Bildung von reaktiven Sauerstoffspezies, wie z. B. Superoxid (O_2_^−^) [18]. Superoxid reagiert wiederum umgehend mit dem Stickstoffmonoxid (NO) unter der Bildung des hochreaktiven Metaboliten Peroxinitrit (ONOO^−^). Dies führt in der Folge zur Abnahme der vaskulären NO^−^-Bioverfügbarkeit, verbunden mit reduzierter Vasodilatation, erhöhter Vasokonstriktion und damit zur Initiierung des atherosklerotischen Prozesses mit einer erhöhten Wahrscheinlichkeit für die Ausbildung von koronarer Herzerkrankung, Herzinsuffizienz, Schlaganfall und Herzrhythmusstörung [19].

**Figure 2:**
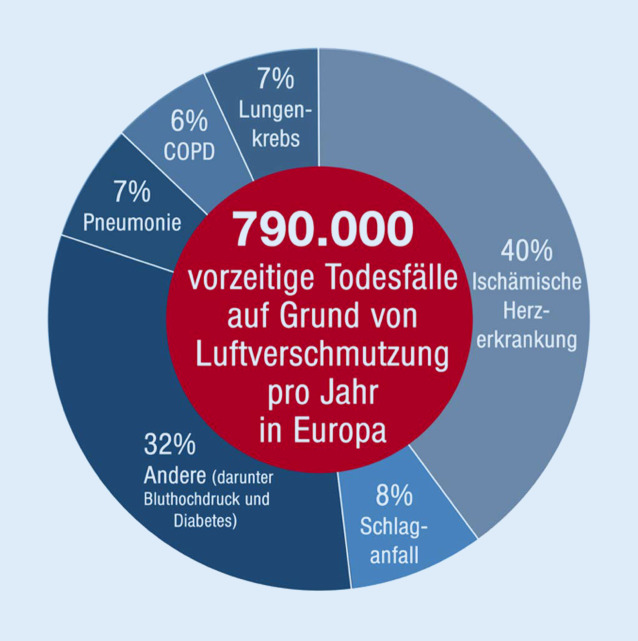

**Figure 3:**
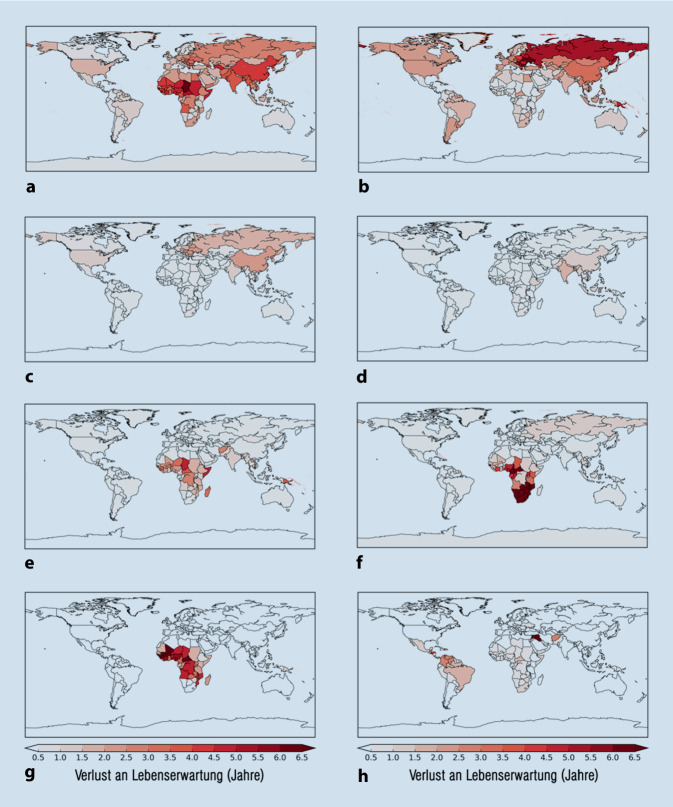

Die Luftverschmutzung hat negative Auswirkungen auf das Gefäßsystem und auf die Lunge [20, 21]. Jüngste Studien haben gezeigt, dass weltweit etwa 8,8 Mio. vorzeitige Todesfälle aufgrund von Luftverschmutzung (PM_2,5_) auftreten. In Europa beziffert sich die Anzahl der vorzeitigen Todesfälle auf etwa 790.000, in erster Linie vermittelt durch Herz-Kreislauf-Erkrankungen wie die ischämische Herzerkrankung (40 %), Schlaganfall (8 %) und weitere nicht übertragbare Erkrankungen wie Bluthochdruck und Diabetes mellitus, die ebenfalls als Folgen von Feinstaub auftreten können (Abb. 2). Knapp mehr als 20 % sind auf Lungenerkrankungen wie Pneumonie (7 %), chronisch obstruktive Bronchitis (6 %) und Lungenkrebs (7 %) zurückzuführen [22]. Die Verschmutzung der Umgebungsluft führt zu einer Verringerung der Lebenserwartung um 2,9 Jahre [23], während das Rauchen als einer der wichtigsten kardiovaskulären Risikofaktoren eine Verringerung der Lebenserwartung um 2,2 Jahre bewirkt, was die bedeutsame Rolle der Luftverschmutzung als Risikofaktor unterstreicht ([23]; Abb. 3).

Diese durch Luftverschmutzung verursachten Erkrankungen entsprechen größtenteils dem Spektrum der Organe, die durch eine COVID-19-Infektion betroffen sind. Dies kann zumindest teilweise erklären, warum eine langjährige, ausgeprägte Exposition mit Luftschadstoffen und daher in der Summe ein bereits vorgeschädigtes Gefäßsystem bzw. eine vorgeschädigte Lunge besonders anfällig sind für zusätzliche Schäden bei COVID-19.

## COVID-19 und Auswirkungen auf das Endothel

**Figure 4:**
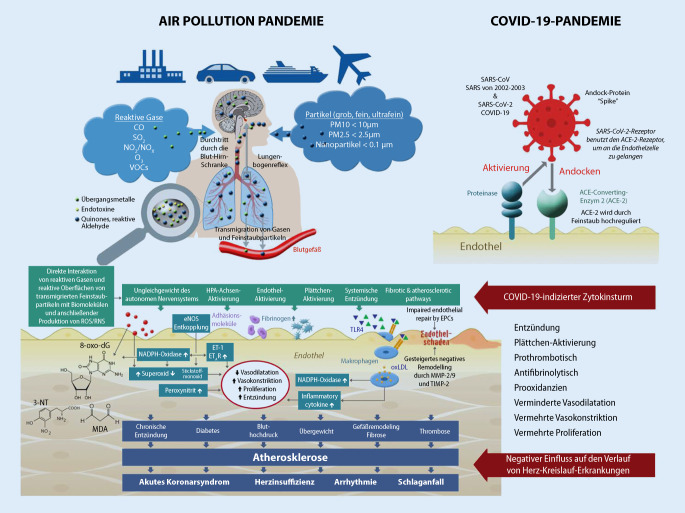

Typische Komplikationen bei COVID-19 sind im kardiovaskulären Bereich ein akutes Koronarsyndrom, Herzinsuffizienz, Herzrhythmusstörung, Thrombose, Lungenembolie und Myokarditis [5]. Weiterhin werden schwer verlaufende Lungenzündungen bis hin zu einem akuten Lungenversagen und der damit erforderlichen extrakorporalen Lungenunterstützung beschrieben [5]. Auf pathophysiologischer Ebene postulierten Peter Libby und Thomas Lüscher kürzlich, dass COVID-19 letztendlich eine Endothelerkrankung darstellen könnte [24]. Das Endothel ist zum einen eine mechanische Barriere zwischen dem Blutstrom und den glatten Muskelzellen und zum anderen ein Organ, das komplexe Funktionen ausübt – wie die Sekretion des Radikals NO für die Vasodilatation, von Endothelin für die Vasokonstriktion sowie von Wachstumsfaktoren und chemotaktischen Faktoren, die strukturelle Veränderungen der Gefäßwand induzieren. Sowohl die Regulation der gesunden als auch der kranken Gefäßwand wird durch die Endothelzellen gesteuert. Weiterhin reguliert das Endothel das Gleichgewicht zwischen Thrombose und Fibrinolyse sowie Antioxidanzien und Prooxidanzien und des Entzündungsgleichgewichts ([24]; Abb. 4).

Im Falle einer Virusinvasion bei COVID-19 nach Einschleusen des Virus in die Zelle via ACE-2-Rezeptoren erlebt die Endothelzelle einen sog. Zytokinsturm, der die Produktion von Prokoagulanzien wie Gewebefaktor, Thromboxan und CD40-Liganden stimuliert und durch Hemmung der Bildung von Plasminogenaktivatorinhibitor Typ 1 (PAI-I) antifibrinolytisch wirkt. Die vermehrt gebildeten reaktiven Sauerstoffspezies (z. B. O_2_) reagieren mit NO unter der Bildung des Metaboliten ONOO^–^, die in der Folge zur Ausbildung einer schweren endothelialen Dysfunktion beitragen [25], d. h. mehr Vasokonstriktion, weniger Vasodilatation und ein prothrombotisches Milieu mit resultierenden schweren kardiovaskulären Komplikationen wie Myokardinfarkt, akute Herzschwäche, Thrombose und schwere Lungenembolien [5].

## Interaktionen von Luftverschmutzung und COVID‑19

Die gesundheitlichen Nebenwirkungen von Luftverschmutzung und einer akuten COVID-19 weisen somit viele Parallelen auf, insbesondere unter dem Aspekt der schweren Schädigung der Gefäßfunktion und der Lungenfunktion. Daher ist es nachvollziehbar, dass insbesondere Patienten mit einer Gefäßfunktionsstörung und bei Anwesenheit kardiovaskulärer Risikofaktoren wie Rauchen, Übergewicht, arterielle Hypertonie und Diabetes mellitus eher gefährdet sind im Rahmen von COVID-19. Gleiches gilt besonders für Patienten mit manifesten Herz-Kreislauf-Erkrankungen wie der koronaren Herzerkrankung, Herzinsuffizienz, Thrombose und Lungenembolie. In Bezug auf die Interaktionen beider Pandemien, der Luftverschmutzungs- und COVID-19-Pandemie, gibt es Berichte wonach insbesondere in der Lunge der ACE-2-Rezeptor durch Feinstaub hochreguliert werden kann. Die Autoren sprechen hier von einem Double-Hit-Konzept, Schädigung der Lunge durch Feinstaub und NO_2_ in Kombination mit einer vermehrten Viruseinschleusung in das Lungenepithel bei COVID-19 aufgrund der Hochregulation des ACE-2-Rezeptors [26]. Eine Arbeitsgruppe aus Italien konnte in Feinstaubpartikeln aus Bergamo in Italien SARS-CoV-2-mRNA nachweisen. Somit kann spekuliert werden, dass der Feinstaub möglicherweise den Träger oder sogar Spreader des Virus darstellen kann [27], wie schon im Rahmen von Influenzaepidemien vermutet wurde [28]. Dieses Konzept muss jedoch in weiteren Studien etabliert werden [28].

## Schlussfolgerungen und klinische Implikationen

Die Ergebnisse der vorgestellten Studien legen nahe, dass die Reduzierung der Luftverschmutzung selbst bei relativ niedrigen Feinstaubkonzentrationen erhebliche Vorteile bringen kann. Die Verbesserung der Expositions-Reaktions-Beziehung und die Verringerung von methodischen Unsicherheiten erfordern zusätzliche Datenanalysen auf Basis großer Kohorten, um den Zusammenhang zwischen Luftverschmutzung und COVID-19 zuverlässiger zu bestimmen. Umweltperspektivisch kann im Rahmen der COVID-19-Pandemie festgehalten werden, dass das Streben nach wirksamen Maßnahmen zur Reduzierung anthropogener Emissionen, die sowohl Luftverschmutzung als auch den Klimawandel verursachen, beschleunigt werden sollte. Die COVID-19-Pandemie wird mit der Impfung der Bevölkerung oder mit der Herdenimmunität durch weitreichende Infektion der Bevölkerung begrenzt werden können. Es gibt jedoch keine Impfstoffe gegen schlechte Luftqualität und den Klimawandel. Daher stellt die Emissionsminderung ein wichtiges Ziel dar. Der Übergang zu einer grünen Wirtschaft mit sauberen, erneuerbaren Energiequellen wird sowohl die Umwelt als auch die öffentliche Gesundheit lokal durch eine verbesserte Luftqualität und global durch die Begrenzung des Klimawandels fördern.

## Fazit für die Praxis

- Ergebnisse aus der ersten SARS-Pandemie und zu Beginn der COVID-19-Pandemie zeigten, dass die Sterblichkeit v. a. in Regionen mit stärkerer Luftverschmutzung erhöht ist.
- Aktuelle Untersuchungen belegen, dass etwa 15 % der weltweiten COVID-19-Todesfälle auf Luftverschmutzung zurückzuführen sind. Der Anteil der luftverschmutzungsbedingten COVID-19-Todesfälle in Europa liegt bei 19 %, in Nordamerika bei 17 % und in Ostasien bei 27 %.
- Luftverschmutzung und COVID-19 führen zu ähnlichen Schäden für das kardiopulmonale System, die möglicherweise den Zusammenhang zwischen Luftverschmutzung und erhöhter COVID-19-Mortalität erklären.
- Folglich kann eine langjährige, ausgeprägte Exposition mit Luftschadstoffen und daher in der Summe ein bereits vorgeschädigtes Gefäßsystem bzw. eine vorgeschädigte Lunge besonders anfällig sein für zusätzliche Schäden bei COVID-19.
- Maßnahmen zur Reduzierung anthropogener Emissionen, die sowohl Luftverschmutzung als auch den Klimawandel verursachen, sollten beschleunigt werden.
